# Cancer cell-specific mitochondrial reactive oxygen species promote non-heme iron uptake and enhance the proliferation of gastric epithelial cancer cell

**DOI:** 10.3164/jcbn.17-8

**Published:** 2017-09-26

**Authors:** Hiromu Ito, Hiromi Kurokawa, Aki Hirayama, Hiroko P. Indo, Hideyuki J. Majima, Hirofumi Matsui

**Affiliations:** 1 Faculty of Medicine, University of Tsukuba, 1-1-1 Tennodai, Tsukuba 305-8575, Japan; 2 Center for Integrative Medicine, Tsukuba University of Technology, 4-12-7 Kasuga, Tsukuba 305-8521, Japan; 3 Graduate School of Medical and Dental Sciences, Kagoshima University, 8-35-1 Sakuragaoka, Kagoshima 890-8544, Japan; 4 Kyoto Prefectural University of Medicine, Kamigyo-ku, Kyoto 602-8566, Japan

**Keywords:** non-heme iron, mitROS, gastric epithelial cell, divalent metal transporter 1, ferroportin

## Abstract

Iron is an essential nutrient for life and is involved in many important processes such as oxygen transport and DNA synthesis. However, excess amounts of iron can cause carcinogenesis by producing reactive oxygen species. Thus, the cellular transport of iron must be tightly regulated. In the human body, iron may be present as heme or non-heme iron. The mechanisms governing the cellular transport of these forms have not been clearly elucidated. We previously reported that the expression of an important heme transporter, heme carrier protein 1 was regulated by cancer-specific reactive oxygen species derived from mitochondria. In this study, we have asked if mitochondrial reactive oxygen species may also be related with non-heme iron transport. In order to address this question, we have investigated the relationship between mitochondrial reactive oxygen species and accumulation of cellular non-heme iron in a rat gastric normal, cancer and manganese superoxide dismutase-overexpressing cancer cell line, in which reactive oxygen species from mitochondria are specifically scavenged. We have also analyzed the expression of divalent metal transporter 1 and ferroprotin, involved in the incorporation and excretion of non-heme iron, respectively, as well as a hypoxia-related transcription factor HIF-1α, to elucidate the molecular mechanism of non-heme iron accumulation.

## Introduction

For most living organisms, including humans, iron is an essential element known to participate in important processes such as transport of oxygen, DNA synthesis, and cell growth and division.^(1–3)^ Iron deficiency is associated with diseases like anemia in humans, whereas excess iron is associated with neurodegenerative disease and vascular injury, emphasizing the importance of iron homeostasis.^(4,5)^

In recent years, excessive accumulation of iron in tissues has been correlated with carcinogenesis possibly mediated by the Fenton reaction. The Fenton reaction is an iron-specific chemical reaction in which iron (in the form of ferrous ions) reacts with hydrogen peroxide and produces hydroxyl radicals, a highly reactive form of oxygen known to cause cellular damage.^(6,7)^ Reactive oxygen species (ROS), including hydroxyl radicals, cause lipid peroxidation, oxidation of proteins, and DNA injury, ultimately lead to pathology and carcinogenesis.^(8,9)^ In support of the above association between iron accumulation and carcinogenesis, iron stored in the liver in the form of ferritin shows a marked association with hepatocellular carcinoma.^(10,11)^ Despite this, however, there is a dearth of information regarding the cellular transport of iron.

There are two forms of iron in the human body, namely, heme and non-heme iron. Heme is composed of a porphyrin ring that chelates iron, whereas non-heme iron lacks a porphyrin ring. Recently, it has been demonstrated that heme iron is transported by heme carrier protein 1 (HCP1), coded by *SLC46A1*, and is highly expressed in the duodenum.^(12)^ On the other hand, non-heme iron is transported by divalent metal transporter 1 (DMT1) and is exported to the blood stream by ferroportin (FPN), coded by *SLC40A1*. FPN levels are regulated by a peptide, hepcidin, that binds to FPN when intracellular and circulating iron levels are high, leading to its degradation.^(6,13,14)^

Mitochondria aerobically generate ATP, the primary energy source for the cell, but are also the main source of ROS. In cancer cells, the electron transport chain is often disrupted because of mitochondrial dysfunction. Consequently, in these cells, electrons leak and react with oxygen, increasing superoxide-anion generation.^(15)^ This in turn results in higher ROS levels in cancer cells than in normal cells. Elevated ROS levels trigger a variety of signal transduction pathways and induce the expression and activation of several genes and proteins.^(16)^ Therefore, it is possible to envisage that intracellular ROS, especially mitochondrial ROS (mitROS), modulate the transport of both heme and non-heme iron in cancer cells.

We previously reported that the expression of the HCP1 heme iron transporter was enhanced in cancer cells and was controlled by mitROS.^(17)^ However, the detailed mechanism of non-heme iron transport and its association with mitROS are as yet uninvestigated. In this study, we have elucidated a relationship between non-heme iron transport and mitROS by studying the influence of non-heme iron incorporated by a normal rat gastric mucosal cell line, its cancer-like derivative, and its cancer-like derivative overexpressing manganese superoxide dismutase (MnSOD), a superoxide-scavenging enzyme that expresses in the mitochondrion specifically, facilitating the study of the effect of mitochondrion-derived ROS.^(18)^

## Materials and Methods

### Cell lines

We established and used four types of gastric mucosal cell lines: a normal rat cell line, RGM1, its cancer-like mutated cell line, RGK1, a MnSOD-overexpressing RGK cell line, RGK MnSOD, and the RGK cell line transfected with a plasmid without the MnSOD cDNA (vector alone), RGK vector.^(19,20)^ As mentioned above, MnSOD expresses in mitochondria and can scavenge ROS from mitochondria specifically.^(21)^

### Cell culture

RGM1 was cultured in DMEM/F12 with l-glutamine (Life Technologies Co., Carlsbad, CA) and RGK cell types were cultured in DMEM/F12 without l-glutamine. These media included 10% inactivated fetal bovine serum (Biowest LLC, Kansas City, MS) and 1% penicillin and streptomycin (Life Technologies, Co.). All cells were cultured in atmospheric air with 5% CO_2_ at 37°C.

### Cellular uptake study of non-heme iron

The amounts of non-heme iron incorporated in each cell line were determined using radioactive iron chloride (PerkinElmer Japan, Co., Ltd., Kanagawa, Japan). Cells were seeded in a 12-well cell culture plate at a density of 1 × 10^5^ cells/well and incubated overnight. After removing medium, the cells were incubated in the iron incubation buffer [25 mM Tris, 25 mM 4-morpholineethanesulfonic acid (MES), 140 mM NaCl, 5.4 mM KCl, 5 mM glucose, 1.8 mM CaCl_2_, 0.44 mM sodium ascorbate, pH 6.0] containing 0.3 µM ^59^FeCl_3_ and 3 µM ^56^FeSO_4_ for 10, 30 and 60 min at 37°C. The reduction of ^59^Fe(III) to ^59^Fe(II) was achieved by ^56^FeSO_4_ in the incubation buffer.^(22)^ The radioactive iron uptake was terminated by washing the cells with PBS thrice. Cells were detached by trypsin-EDTA treatment, and the amount of radioactive iron in the cells was measured in a gamma counter (AccuFLEX γ7000, Hitachi Aloka Medical, Ltd., Tokyo, Japan).

### Western blot analysis

Western blot analysis of DMT1, FPN, and hypoxia inducible factor 1 alpha subunit (HIF-1α) was performed as previously reported.^(23)^ Cell lysates from each cell line were prepared using NuPAGE LDS Sample buffer (Life Technologies Co.) and heated to 70°C for 15 min. Lysates (10 µl each) were applied to each well of NuPAGE Novex 12% Bis-Tris gels (Life Technologies Co.) for SDS-polyacrylamide gel electrophoresis (SDS-PAGE). The gel was electrophoresed at 100 V for 1.5 h, and the electrophoresed proteins were transferred to a polyvinylidene difluoride (PVDF) membrane (Millipore Co., Billerica, MA) by applying a current of 1.2 mA/cm^2^ for 1 h. The membrane was then immersed in blocking reagent (TOYOBO CO., LTD., Osaka, Japan) for 1 h, and was washed thrice with PBS-T (Phosphate buffered saline with 0.1% Tween 20). Blocked membrane was then incubated with anti-DMT1 (ab140977), anti-SLC40A1 (ab78066) (Abcam plc., Cambridge, UK) or anti-HIF-1α (#14179) (Cell Signaling Technology Japan, K.K.) antibody in Can Get Signal Immunoreaction Enhancer Solution 1 (TOYOBO CO., LTD., Osaka, Japan) (1:1,000) overnight. The membrane was removed from the primary antibody solution, washed with PBS-T thrice, and then incubated with horseradish peroxidase (HRP)-linked anti-rabbit IgG antibody (#7074) (Cell Signaling Technology Japan, K.K) in Can Get Signal Immunoreaction Enhancer Solution 2 (TOYOBO) (1:1,000) for 2 h. The membrane was washed with PBS-T thrice and was immersed in Lumina forte western HRP substrate (Millipore Co.). Luminescence from the membrane was observed by LAS4000 (GE Health Care Japan, Tokyo, Japan). As a loading control, β-actin was detected with β-actin antibody (#4967) (Cell Signaling Technology Japan, K.K). The protein expression levels were quantified using ImageJ software (National Institutes of Health, Bethesda, MA).

### Measurement of ROS generation by electron spin resonance

Intracellular ROS generation after iron treatment was detected by electron spin resonance (ESR) according to a previous study.^(24)^ Briefly, cells were seeded on a sterilized glass cover slide (49 × 5 × 0.2 mm) at confluency and incubated overnight. Cells were then exposed to fresh medium containing 500 µM FeSO_4_ for 1 h and then immersed in the respiratory buffer containing 5 mM succinate (Sigma-Aldrich Japan K.K., Tokyo, Japan), 5 mM malate (Wako), 5 mM glutamate (Sigma-Aldrich Japan K.K.), 5 mM nicotinamide adenine dinucleotide (NADH) (Sigma-Aldrich Japan K.K.) and 5 µl 5,5-dimethyl-1-pyrroline-*N*-oxide (DMPO) (Dojindo Laboratories, Kumamoto, Japan). The slide was set in a tissue glass, and ESR spectra were obtained using a JEOL-TE X-band spectrometer (JEOL, Ltd., Tokyo, Japan) using the following measurement conditions: 10 mW incident microwave power, 9.4 GHz frequency, and 0.1 mT field modulation amplitude. The experiments were performed three times independently.

### Cell proliferation assay

The effect of iron on cellular proliferation was investigated using the Cell Counting Kit 8 (Dojindo Laboratories), a water-soluble tetrazolium (WST)-8 based colorimetric assay. Cells were seeded in a 96-well cell culture plate at a density of 1 × 10^4^ cells/well and incubated overnight. They were then incubated in 100 µl iron incubation buffer containing 0, 1, 2, 5, 10, 20, 50 or 100 µM FeSO_4_ for 6 h at 37°C. After incubation, cells were washed twice with PBS and the medium was replaced with fresh medium containing 10% Cell Counting Kit 8 reagent and further incubated. The absorbance at 450 nm was measured using a DTX880 multi-mode micro-plate reader (Beckman Coulter Inc., Brea, CA).

### Statistical analysis

All data are expressed as mean ± SD. Statistical analysis was performed using SPSS statistics 21 software (IBM Corporation, NY). Tukey’s test or Games-Howell’s test was used for comparison of more than two data sets and Student’s *t* test for comparison of two data sets; *p*<0.01 and *p*<0.05 were considered statistically significant.

## Results

### Specific accumulation of non-heme iron in cancer cells

**Fig. 1:**
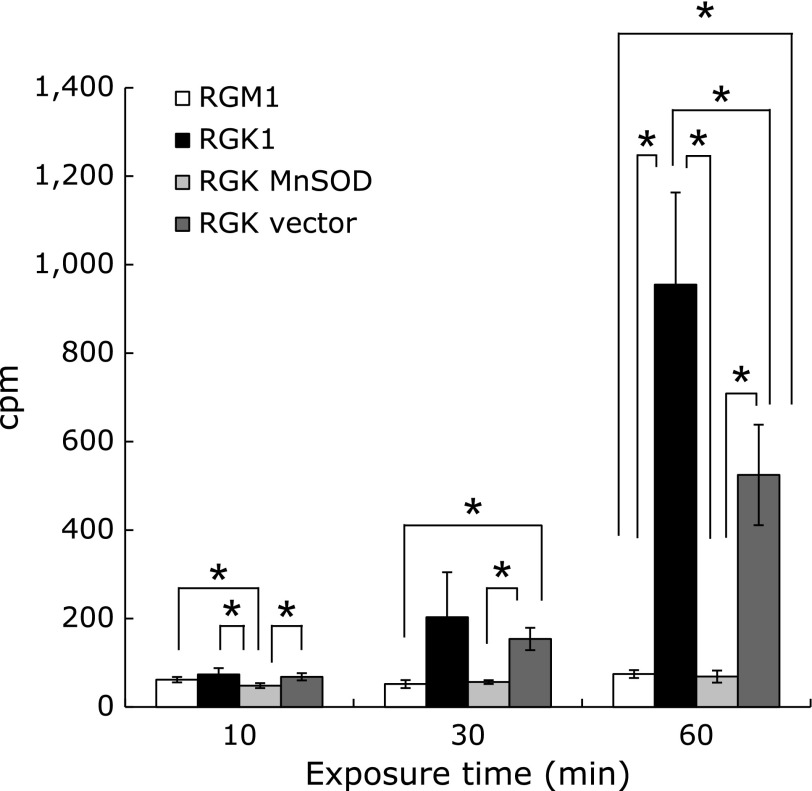
The results of cellular uptake levels of iron in each cell line were shown. Cells were exposed to radioactive iron (^59^Fe) and uptake of iron was measured by measuring the level of radioactivity in cell lysates. Statistical significance was tested by the Games-Howell test. *n* = 6, error bar: SD; *p<0.05.

We compared the incorporation of non-heme iron among normal cells (RGM1), cancer cells (RGK1), and MnSOD-overexpressing (RGK MnSOD) and vector-control (RGK vector) cancer cells by measuring the amount of radioactive iron present in lysates made from each cell line cultured in the presence of ^59^Fe. RGK1 and RGK vector-control cells showed a dramatic increase in the accumulation of radioactive iron in a time-dependent manner, whereas RGM1 and RGK MnSOD cells did so only marginally (Fig. 1). Cancer cells showed significantly greater accumulation of radioactive iron than normal cells did, particularly at 60 min. Importantly, MnSOD-overexpression attenuated iron uptake, indicating that intracellular mitROS were indeed associated with cellular uptake of non-heme iron.

### Expression of iron transport proteins and hypoxia transcription factor in cells

**Fig. 2:**
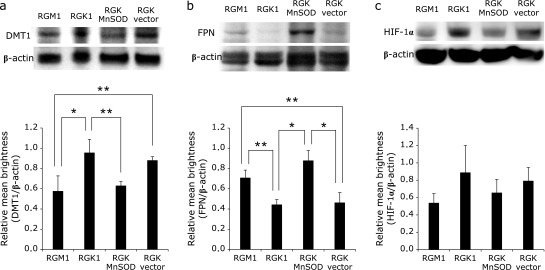
The results of western blotting analyses of iron transport proteins and a hypoxia inducible factor protein. Expression of iron transporters divalent metal transporter 1 (DMT1) (a), ferroportin (FPN) (b) and a hypoxia-related transcription protein HIF-1α (c) were quantified and compared among cell lines by western blot analysis. The levels of β-actin were used as normalizing control. The experiments were performed thrice and statistical significance was tested by the Tukey HSD test. *n* = 3, error bar: SD; *p<0.01, **p<0.05.

Next, we compared the expression levels of the DMT1 and FPN iron transporters in the above cells by western blot analysis. DMT1, involved in iron uptake, was expressed to a higher level in cancer cells than in normal and MnSOD-overexpressing cancer cells (Fig. 2a). In contrast, the iron excretion protein FPN showed lower expression in cancer cells than in normal and MnSOD-overexpressing cells (Fig. 2b). These data suggest a tendency of cancer cells to retain and actively take up iron, in contrast to normal cells, and are in agreement with the greater uptake of extracellular iron by cancer cells (Fig. 1). Additionally, the expression of HIF-1α was also higher in RGK1 cells and decreased in RGK MnSOD cells (Fig. 2c) in line with DMT1 expression levels in these cells.

### Iron exposure-induced ROS generation in cancer cells

**Fig. 3:**
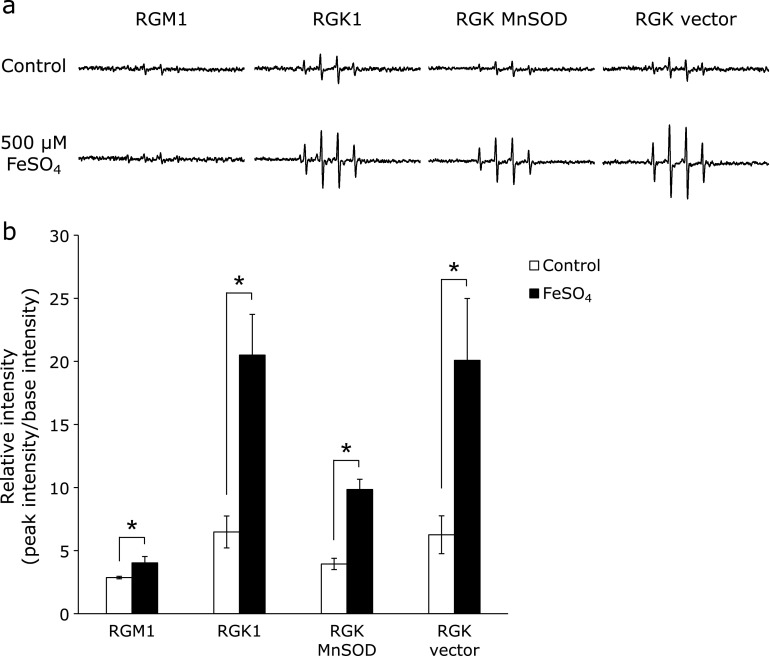
The results of electron spin resonance (ESR) of living cells after iron treatment. (a) ESR spectra of each cell line with or without 500 µM FeSO_4_ treatment for 1 h were shown. Cellular ROS were trapped using 5,5-dimethyl-1-pyrroline 1-oxide (DMPO), and the signals of DMPO-adducts were detected. (b) Relative ESR intensity in each cell line was calculated. Statistical significance was tested using Student’s *t* test. *n* = 3, error bar: SD; *p<0.05.

Given the increased iron levels in cancer cells, we measured intracellular ROS levels using ESR in cells treated with 500 µM FeSO_4_. Fig. 3a showed ROS levels were significantly higher in RGK1 and vector cells after treatment of iron, whereas ESR peaks were slightly detectable in RGM1 cells. In RGK MnSOD cells, weak peaks were observed without iron treatment and signal increase was observed after FeSO_4_ treatment. In all of cell lines, relative signal intensities were increased by iron treatment, however, the increase level was suppressed by overexpression of MnSOD in cancer cells. In addition, the increase of peak intensity in normal cells was lower than other cancer cell lines (Fig. 3b). These results indicated that iron treatment induced cancer cell-specific enhancement in ROS generation and that the ROS were primarily derived from mitochondria because MnSOD expresses in mitochondria specifically and overexpression of MnSOD suppressed the increase of ROS generation in cancer cells.

### Enhancement of cancer cellular proliferation

We next attempted to determine whether the incorporation of iron had any influence on cellular proliferation. In normal (RGM1) cells, cellular proliferation was not induced, even at FeSO_4_ concentrations of up to 50 µM, and at 100 µM, the cell viability was lost. However, in all the other cell lines, proliferation increased with extracellular iron in a dose-dependent manner and, unlike in RGM1 cells, loss of viability was not observed even at 100 µM iron exposure. In particular, RGK1 cells proliferated dramatically upon iron treatment and their proliferation was significantly greater than that of normal cells at iron concentrations greater than 20 µM. Further, MnSOD overproduction suppressed proliferation potential to some extent, indicating that exposure to non-heme iron may promote cancer cell-specific proliferation.

## Discussion

We have previously reported that cancer cells produce larger amounts of ROS than normal cells do, and MnSOD expression in cancer cells suppress ROS production.^(24)^ Since MnSOD is specifically able to scavenge ROS derived from mitochondria, using MnSOD overexpressing cancer cells (RGK MnSOD) allows direct examination of the influence of mitROS on iron uptake and cell proliferation in cancer cells. Our study demonstrates the importance of mitROS in the regulation of cancer cell-specific iron uptake. Additionally, in this study, we have demonstrated for the first time that mitROS play an important role in the incorporation of non-heme iron in gastric epithelial cells.

Our study establishes an important molecular link between iron uptake and mitROS in cancer cells. As seen in Fig. 1, RGK1 and RGK vector cells accumulate radioactive iron in a time-dependent manner, whereas RGK MnSOD cells do so to a much lower extent. RGM1 cells also showed much lower iron accumulation compared to RGK1 cells. Additionally, the expression level of DMT1 in RGK1 was higher than that in RGK MnSOD and RGM1 cells, whereas FPN expression was higher in RGK MnSOD cells than in RGK1 cells (Fig. 2). These data indicate that mitROS influence the expression level of the non-heme iron incorporation protein DMT1 and its excretion protein FPN, and that these proteins in turn regulate the levels of intracellular iron. Thus, although RGK1 cancer cells are poised to incorporate and accumulate non-heme iron, RGM1 normal cells and RGK MnSOD cells are unable to incorporate large amounts of non-heme iron and indeed may readily allow its excretion.

The expression of iron uptake proteins has been reported to be related to hypoxia.^(25)^ In hypoxic environments, HIF, which is a transcription factor, is stabilized and activates the transcription of various genes involved in iron metabolism such as erythropoietin and heme oxygenase-1.^(26,27)^ There are several types in HIFs reported, such as HIF-1α and HIF-2α, which are subunits of the HIF-1 and HIF-2 complexes, and they induce transcription by interacting with hypoxia response elements.^(28)^ Qian *et al.*^(29)^ reported that HIF-1 stabilization alone is not adequate to induce iron incorporation via DMT1 upregulation. On the other hand, Mastrogiannaki *et al.*^(30)^ reported that HIF-2α, but not HIF-1α, regulated the expression of DMT1. The activation of HIFs is known to be linked to ROS, and mitROS in particular seem to play an important role in this process.^(31–34)^ Therefore, we propose that stabilization of HIFs by mitROS may regulate the expression of DMT1, and promote cellular uptake of non-heme iron. This hypothesis is supported by the observation that scavenging cancer cell-specific mitROS led to suppression of DMT1 expression. Indeed, HIF-1α levels in RGK1 cells were also much higher than in MnSOD overexpressing cells (Fig. 2c). Additionally, mitROS also appear to inhibit the expression of FPN. Hepcidin, a peptide hormone, is known to bind to FPN, causing the latter to be degraded.^(35)^ Hepcidin is induced not only in response to increased levels of intracellular iron but also by the action of proinflammatory cytokines such as interleukin-6 (IL-6).^(36)^ Besides, although hepcidin is produced mainly in the liver, it is upregulated in gastric parietal cells by IL-6 or infection of *Helicobacter pylori*, and mitROS is already implicated in this process.^(37–39)^ Thus, enhancement of hepcidin production due to mitROS may explain the suppression of FPN expression in cancer cells, allowing reduced iron efflux. Alternatively, hepcidin-independent regulation of FPN levels may be mediated by HIF-2α as demonstrated by Taylor *et al.*^(40)^

**Fig. 4:**
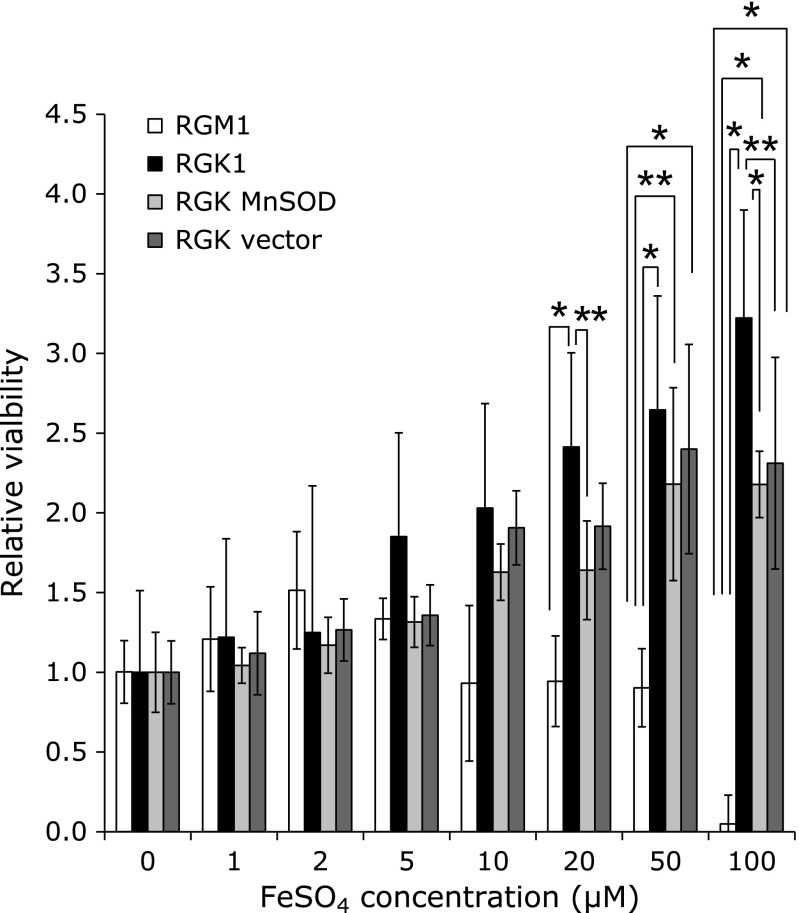
The result of cell viability tests after iron treatment. Cellular proliferation rates after exposure to FeSO_4_ at the indicated concentration were measured using the WST-8 colorimetric assay and normalized to values for untreated cells. Statistical significance was tested using the Tukey HSD test. *n* = 6, error bar: SD; *p<0.01, **p<0.05.

Our results demonstrate that iron accelerated mitROS production and proliferation in cancer cells but not in normal cells (Fig. 3 and 4). Excess iron is known to damage mitochondrial DNA and cause respiratory dysfunction in normal cells, and intracellular iron is associated with cancer cell proliferation.^(41,42)^ Indeed, decrease of iron levels by phlebotomy can reduce the risk of hepatocellular carcinogenesis in patients with chronic hepatitis C.^(43)^ Further, Chen *et al.*^(44)^ have reported that lower expression of FPN enhances cellular proliferation and causes poor prognosis in breast cancer. It appears then, that an overload of iron is likely to enhance the “cancerous” characteristics of cancer cells by enhancing their proliferative potential. Since MnSOD overexpression attenuated ROS production and proliferation of cancer cells in response to exposure to iron, we suggest that the signaling pathway triggered by mitROS may reinforce the proliferative properties of cancer cells.

In conclusion, we have shown that cancer cells incorporate larger amounts of non-heme iron than normal cells do and that this phenomenon may be attributed to the enhancement of expression of iron transport protein DMT1 by mitROS. Intracellular overload of iron in turn may induce cancer cells to produce excess ROS and hence promote cancer-cell specific proliferation. These results may be associated with ROS-related disease and bring some adverse effects to patients with disease such as inflammation and cancer.
